# SANS FLUORO Optimized: A Case Report of Pulmonary Vein Isolation in a Patient with Cardiac Resynchronization Therapy Defibrillator and the Optimizer™ III Device

**DOI:** 10.19102/icrm.2020.110502

**Published:** 2020-05-15

**Authors:** Hyacinth C. Percell, Lynn Saeger, Erin Sharpe, Rose Saalfeld, Robert L. Percell

**Affiliations:** ^1^SANS FLUORO Association, Electrophysiology Section, Bryan Heart Institute, Lincoln, NE, USA; ^2^Electrophysiology Section, Bryan Heart Institute, Lincoln, NE, USA

**Keywords:** Atrial fibrillation, congestive heart failure, implantable cardioverter-defibrillator, Optimizer, pulmonary vein isolation

## Abstract

We offer the first reported case of a pulmonary vein isolation (PVI) procedure performed in a patient with two devices, specifically a cardiac resynchronization therapy defibrillator (CRT-D) and an Optimizer™ III device (Impulse Dynamics, Mount Laurel, NJ, USA), using the SANS FLUORO technique with zero fluoroscopy. In total, this patient had six leads traversing the right atrium, including two right atrial leads, three right ventricular leads—two associated with the Optimizer™ system and one implantable cardiac defibrillator lead—and a left ventricular lead.

## Introduction

We report the first-ever pulmonary vein isolation (PVI) procedure performed in a patient with two devices, a cardiac resynchronization therapy defibrillator (CRT-D) and an Optimizer™ III device (Impulse Dynamics, Orangeburg, NY, USA) using the SANS FLUORO technique (zero fluoroscopy). This patient had a total of six leads traversing the right atrium: two right atrial (RA) leads, three right ventricular (RV) leads (two for the Optimizer™ III device and one implantable cardioverter-defibrillator lead), and a left ventricular (LV) lead.

## Case presentation

The patient was a 69-year-old male with an implantable cardiac monitor, left bundle branch block with an ejection fraction of 25%, and a CRT-D device. He had previously undergone successful implantation of the Optimizer™ III device (Impulse Dynamics, Orangeburg, NY, USA) as part of a clinical trial. Subsequently, he developed severely symptomatic atrial fibrillation (AF) requiring multiple cardioversions.

### Procedure

The patient underwent uncomplicated PVI using radiofrequency (RF) via the SANS FLUORO technique. Briefly, groin access was obtained with 7-, 8-, and 9-French (Fr) short sheaths in the right groin. Coronary sinus catheter (Inquiry™; Abbott Laboratories, Chicago, IL, USA), intracardiac echocardiography (ICE) catheter (8-Fr ACUSON Acunav^®^; Siemens, Berlin, Germany) were adopted, while single transseptal puncture was performed with a long guide catheter (Preface^®^; Biosense Webster, Diamond Bar, CA, USA) and a HEARTSPAN (Biosense Webster, Diamond Bar, CA, USA) transseptal needle. Right and left atrial mapping was completed with a multielectrode (Advisor HD GRID^®^; Abbott Laboratories, Chicago, IL, USA) using the EnSite Precision system (Abbott Laboratories, Chicago, IL, USA). PVI was performed with an irrigated contact force-sensing catheter (TactiCath™; Abbott Laboratories, Chicago, IL, USA). Esophageal temperature probe (Medi-Therm^®^ DP400CE Esophageal/Rectal Temperature Probe; Stryker Medical, Kalamazoo, MI, USA) placement was confirmed with the ICE catheter.

**Figure 1:**
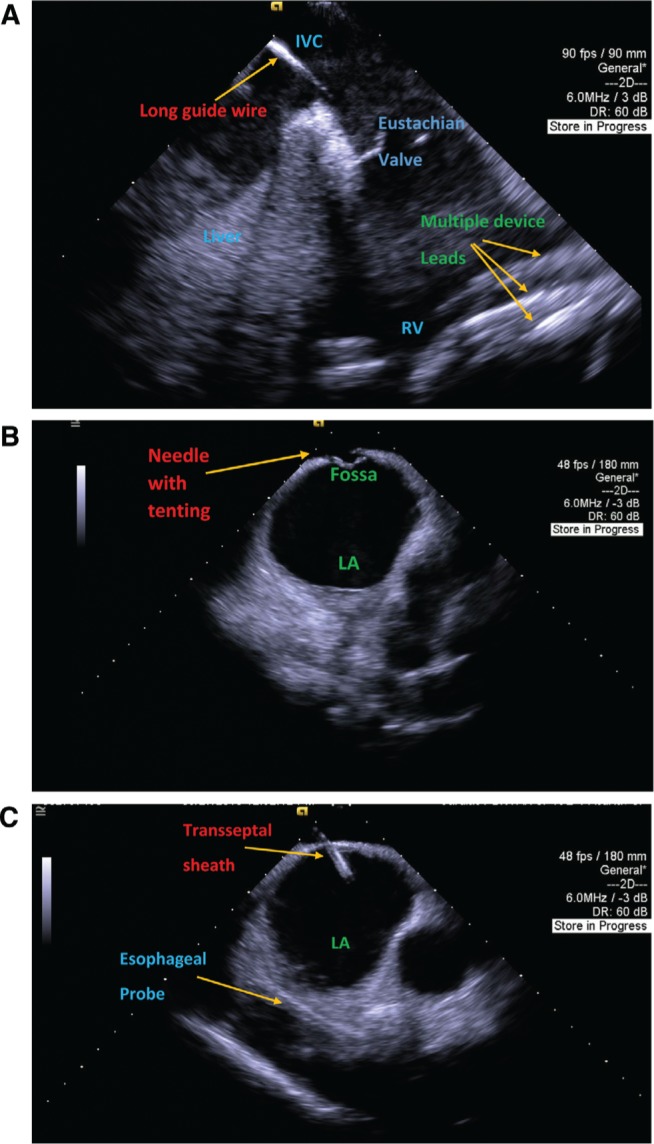
**A:** Several device leads in the RV as well as the long guidewire in the inferior vena cava (IVC). **B:** Transseptal needle with tenting. **C:** Transseptal sheath in the LA as well as the esophageal temperature probe.

**Figure 2:**
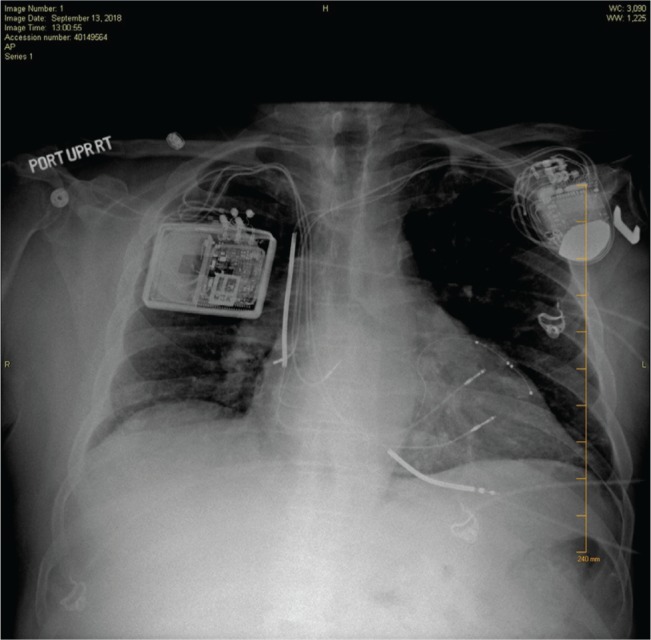
Preprocedure chest radiograph revealing CRT-D and the Optimizer™ III device (Impulse Dynamics, Orangeburg, NY, USA).

**Figure 3:**
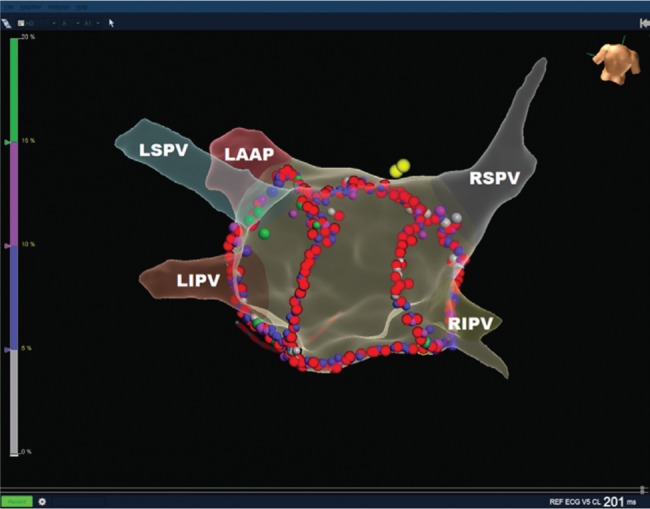
Posterior view of the LA revealing four pulmonary veins and the left atrial appendage. Red tags are lesions with lesion size index values of more than 4.5 on the posterior wall and more than 5.0 anteriorly. White, blue, magenta, and green lesions represent impedance decreases of 5%, 10%, 15%, and 20% in ohms, respectively. Yellow points denote the His in the RA. LSPV: left superior pulmonary vein; LIPV: left inferior pulmonary vein; RSPV: right superior pulmonary vein; RIPV: right inferior pulmonary vein; LAAP: left atrial appendage.

**Figure 4:**
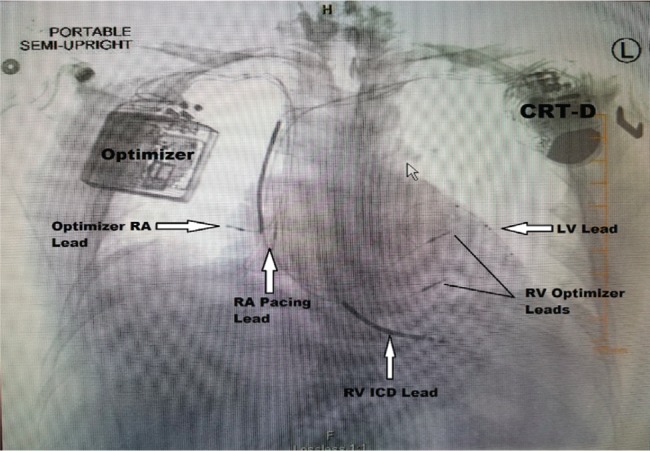
Postprocedure anteroposterior chest radiograph, revealing no change occurred in lead position.

There were no issues with catheter placement or transseptal access **(Figures 1A–1C)**. The preprocedure chest radiograph from a prior hospitalization is shown in **Figure 2**. There was increased scar burden in the left atrium (LA) based on voltage mapping, with ablation lesions showing the left atrial roof and posterior line **(Figure 3)**. Post-PVI, entrance and exit blocks were demonstrated. He had no procedural complications and was discharged the following day. The postprocedure CRT-D check revealed no change in lead sensing, threshold, or impedance values. The postprocedure chest radiograph showed no change in lead position **(Figure 4)**. The patient remained free of AF at his three- and six-month follow-up visits.

## Discussion

We report the first PVI procedure in a drug-refractory, persistent, symptomatic AF patient with six leads in the heart using the SANS FLUORO (zero fluoroscopy) technique.^1^ AF is the most common cardiac arrhythmia and increases dramatically with age. AF frequently coexists with congestive heart failure (CHF), especially in patients with LV dysfunction.^2^ PVI has been shown to be effective in reducing AF burden and improving mortality in patients with LV dysfunction relative to amiodarone.^3^

Most PVI procedures are performed with fluoroscopy, whether involving RF or cryoablation, despite recent studies supporting that zero fluoroscopy techniques are feasible, safe, and well-tolerated with similar rates of efficacy.^4–11^ Fluoroscopic techniques have been shown to increase the risk of a variety of medical maladies including but not limited to cataracts, head and neck tumors, local erythema, skin desquamation, organ atrophy, birth defects, and bone cancer.^12,13^ Lead apron use has contributed to multiple orthopedic problems, with nearly half of cardiologists indicating that they are plagued by back, neck, or leg problems.^14^

The Optimizer™ III (Impulse Dynamics, Orangeburg, NY, USA) is a unique implantable device used in patients with New York Heart Association classes II and III CHF as part of the FIX-HF-5 studies. This rechargeable device delivers impulses to the heart using pacemaker leads during the absolute refractory period in order to modulate cardiac contractility. No mortality benefit has been shown; however; significant improvements in peak ventilatory oxygen uptake and quality of life of patients with moderate to severe heart failure as compared with the effects of the best available medical care were demonstrated.^15,16^

We believe that all ablation procedures should be performed with zero radiation (SANS FLUORO) to protect patients, operators, and electrophysiology staff from the harmful effects of radiation and protective lead use. In our laboratory, these procedures are commonly performed in device patients without complication. This case provides further evidence that fluoroless complex ablation procedures are safe, even in device patients.
